# β_2_-Adrenoceptors activation regulates muscle trophic-related genes following acute resistance exercise in mice

**DOI:** 10.3389/fphys.2024.1268380

**Published:** 2024-01-22

**Authors:** Ronaldo L. Abdalla-Silva, Gustavo O. Zanetti, Natalia Lautherbach, Aline Zanatta Schavinski, Lilian C. Heck, Dawit A. P. Gonçalves, Isis C. Kettelhut, Luiz C. C. Navegantes, Wilian A. Silveira

**Affiliations:** ^1^ Department of Biochemistry, Pharmacology and Physiology, Institute of Biological and Natural Sciences, Federal University of Triângulo Mineiro, Uberaba, Minas Gerais, Brazil; ^2^ Exercise Physiology Laboratory, School of Physical Education, Physiotherapy and Occupational Therapy, Universidade Federal de Minas Gerais, Belo Horizonte, Minas Gerais, Brazil; ^3^ Department of Physiology, Ribeirão Preto Medical School, University of São Paulo, São Paulo, Brazil; ^4^ Department of Biochemistry/Immunology, Ribeirão Preto Medical School, University of São Paulo, São Paulo, Brazil; ^5^ Sports Training Center, School of Physical Education, Physiotherapy and Occupational Therapy, Universidade Federal de Minas Gerais, Belo Horizonte, Minas Gerais, Brazil

**Keywords:** resistance exercise, β2-adrenoceptor, skeletal muscle, myostatin, NR4A3

## Abstract

Resistance exercise (RE) training and pharmacological stimulation of β_2_-Adrenoceptors (β_2_-ARs) alone can promote muscle hypertrophy and prevent muscle atrophy. Although the activation of the sympathetic nervous system (SNS) is a well-established response during RE, the physiological contribution of the endogenous catecholamines and β_2_-ARs to the RE-induced changes on skeletal muscle protein metabolism remains unclear. This study investigated the effects of the β_2_-ARs blockade on the acute molecular responses induced by a single bout of RE in rodent skeletal muscles. Male C57BL6/J mice were subjected to a single bout of progressive RE (until exhaustion) on a vertical ladder under β_2_-AR blockade with ICI 118,551 (ICI; 10 mg kg^-1^, i. p.), or vehicle (sterile saline; 0.9%, i. p.), and the gene expression was analyzed in *gastrocnemius* (GAS) muscles by qPCR. We demonstrated that a single bout of RE acutely increased the circulating levels of stress-associated hormones norepinephrine (NE) and corticosterone (CORT), as well as the muscle phosphorylation levels of AMPK, p38 MAPK and CREB, immediately after the session. The acute increase in the phosphorylation levels of CREB was followed by the upregulation of CREB-target genes *Sik1*, *Ppargc1a* and *Nr4a3* (a central regulator of the acute RE response), 3 h after the RE session. Conversely, β_2_-AR blockade reduced significantly the *Sik1* and *Nr4a3* mRNA levels in muscles of exercised mice. Furthermore, a single bout of RE stimulated the mRNA levels of the atrophic genes *Map1lc3b* and *Gabarapl1* (autophagy-related genes) and *Mstn* (a well-known negative regulator of muscle growth). Unexpectedly, the gene expression of *Igf-1* or *Il-6* were not affected by RE, while the atrophic genes *Murf1/Trim63* and *Atrogin-1/Mafbx32* (ubiquitin-ligases) were increased only in muscles of exercised mice under β_2_-AR blockade. Interestingly, performing a single bout of RE under β_2_-AR blockade increased the mRNA levels of *Mstn* in muscles of exercised mice. These data suggest that β_2_-ARs stimulation during acute RE stimulates the hypertrophic gene *Nr4a3* and prevents the overexpression of atrophic genes such as *Mstn*, *Murf1/Trim63*, and *Atrogin-1/Mafbx32* in the first hours of postexercise recovery, indicating that he SNS may be physiologically important to muscle adaptations in response to resistance training.

## 1 Introduction

Skeletal muscle is the most abundant and plastic tissue in the human body, comprising approximately 40%–50% of total body mass (Sartori et al., 2021). This tissue is fundamental for locomotion, breathing, thermogenesis, energy expenditure, and glycemic control (Pedersen, 2013; Schnyder and Handschin, 2015). Due to these important functions, muscle wasting and weakness have been associated with reduced quality of life and higher mortality risks of all causes, cancer, chronic obstructive pulmonary disease (COPD) and aging (Mathur et al., 2014; Santana et al., 2019; Zhou et al., 2023). Despite these alarming findings, there is no effective pharmacological treatment for preventing muscle atrophy in such conditions.

Resistance exercise (RE) training, on the other hand, may increase muscle mass (hypertrophy) and strength (Hornberger and Farrar, 2004; Ruas et al., 2012) and prevent muscle wasting in catabolic situations, such as cancer, glucocorticoids treatment, and sarcopenia (Kim et al., 2016; Padilha et al., 2021; Testa et al., 2022; Macedo et al., 2023). These long-term adaptations to RE training involve acute and transient changes in mRNA expression of various genes in response to each single bout of exercise, including the muscle-derived myokines insulin-like growth factor 1 (IGF-1), myostatin (MSTN) and interleukin 6 (IL-6), among others (Piccirillo, 2019). Such changes in mRNA expression occur between 3h and 12 h after an exercise bout and result in a gradual modification in protein content and activity (Egan and Zierath, 2013). Ultimately, the transcriptional and post-translational regulation induced by RE leads to muscle growth, when the rate of protein synthesis exceeds protein degradation over time (Phillips et al., 1997), while reduced rates of protein degradation seem to be necessary for attenuating muscle atrophy (Graham et al., 2021). Although the health benefits of regular RE training on skeletal muscle physiology are well established, the acute molecular mechanism and signaling pathways controlling protein metabolism after a single bout of RE remains elusive.

Most physiological systems of the body (i.e., nervous, cardiovascular, respiratory, endocrine and musculoskeletal) are stimulated during RE (Athanasiou et al., 2023). For example, the RE acutely increases the secretion of catabolic hormones such as glucocorticoids (e.g., cortisol in humans; corticosterone in rodents) and reduces the anabolic hormone insulin (INS) (Kraemer and Ratamess, 2005; Athanasiou et al., 2023), extracellular alterations associated with decreased protein kinase B (PKB)/Akt and mitogen-activated protein kinases (MAPKs) ERK1/2 phosphorylation/activation in skeletal muscle (Osman et al., 2000; Yang et al., 2008; Hu et al., 2009). On one hand, the dephosphorylation of these intracellular mediators of the INS can activate the Forkhead box class O (*i.e.,* FoxO), leading to transcription of several components of the ubiquitin-proteasome (UPS; *Atrogin-1/Mafbx32* and *MuRF1/Trim63*) and autophagic-lysosomal (ALS; *Map1lc3b* and *Gabarapl1*) systems, which degrade most cellular proteins and organelles in skeletal muscle (Yang et al., 2008; Milan et al., 2015). Accordingly, the rate of protein degradation increases immediately after the cessation of an acute RE session, probably to prevent the accumulation of damaged proteins and organelles (Phillips et al., 1997; Grumati et al., 2011; Aweida and Cohen, 2021). On the other hand, decreased Akt and ERK1/2 phosphorylation and their downstream targets GSK-3 and mTOR activities are associated with reduced protein synthesis (Rommel et al., 2001; Figueiredo and Markworth, 2015; Miyazaki and Takemasa, 2017). Despite that, enhanced myofibrillar and mitochondrial protein synthesis appear to be a critical step in the recovery of muscle homeostasis in the hours after acute RE (Wilkinson et al., 2008; Egan and Zierath, 2013). Even though the molecular mechanisms involved in the increase of the protein synthesis are well characterized, the role of the proteolytic systems after a single bout of RE and during adaptive muscle hypertrophy is not completely understood.

As a physiological stress, RE also stimulates the activity of the sympathetic nervous system (SNS), a branch of the autonomic nervous system (ANS), to meet the metabolic demand of active skeletal muscles during exercise (Athanasiou et al., 2023). Indeed, RE stimulates the sympathoadrenal axis to increase the plasma catecholamines levels (epinephrine [EPI] and norepinephrine [NE]) as a function of exercise intensity (Kraemer and Ratamess, 2005; Athanasiou et al., 2023), which return to the basal levels between 5 and 15 min after the end of exercise (Goto et al., 2008; Fatouros et al., 2010). Moreover, it has been shown that muscle sympathetic nerve activity (MSNA) also increases in proportion to the rise in intensity (Katayama and Saito, 2019). Based on that, the adrenergic actions of SNS can be mediated by a hormonal (catecholamines released from adrenal medulla) and/or a neural mechanism (NE secreted from sympathetic nerve ends) in many tissues, including skeletal muscle (Graça et al., 2013; Khan et al., 2016; Rodrigues et al., 2019).

In skeletal muscle, there is a significant proportion of GTP-binding protein stimulatory (Gαs)-coupled *ß*-adrenoceptors (β-ARs), predominantly of β_2_-subtype (β_2_-AR; ∼90%), that can be activated by endogenous catecholamines via circulation and/or direct muscle noradrenergic innervation (Elfellah et al., 1989; Lynch and Ryall, 2008; Khan et al., 2016). Upon binding of catecholamines, Gαs-coupled β_2_-ARs activate adenylyl cyclase (AC), leading to the increase in intracellular second messenger cyclic adenosine monophosphate (cAMP), and subsequent activation of cAMP-dependent protein kinase (PKA) (Lynch and Ryall, 2008). Once activated, the free catalytic subunits of PKA phosphorylate several substrates, including regulatory enzymes (*i.e.,* glycogen synthetase and hormone-sensitive lipase), the ryanodine receptor (RyR), and the cAMP response element-binding protein (CREB) transcription factor, resulting in enhanced catabolism of energy substrates, muscle contractility, and gene expression, respectively (Altarejos and Montminy, 2011; Berdeaux and Stewart, 2012; Cairns and Borrani, 2015). In contrast to these acute effects of catecholamines, chronic treatment with β_2_-AR agonists promotes muscle growth and can be used for attenuating muscle wasting (Kim and Sainz, 1992; Mersmann, 1998; Yimlamai et al., 2005; Harcourt et al., 2007; Ryall et al., 2007). The molecular mechanisms by which β_2_-AR agonists induce these anabolic and anti-catabolic effects on skeletal muscle are not completely understood but may involve canonical (i.e., cAMP/PKA) and/or non-canonic signaling pathways (Lynch and Ryall, 2008; Gonçalves et al., 2012; Silveira et al., 2020). Indeed, it has been postulated that β_2_-AR agonists stimulate muscle hypertrophy and prevent muscle atrophy by activating Akt/mTOR and Akt/FoxO pathways, respectively, via the subunits βγ of GTP-binding inhibitory protein-coupled β_2_-AR (Giα-Gβγ) (Xiao, 2001; Kline et al., 2007; Lynch and Ryall, 2008; Koopman et al., 2010). Moreover, β_2_AR-induced skeletal muscle growth and strength might also be mediated by *ß*-arrestin 1, a multifunctional adaptor protein that function as a signal transducer required for the activation of other signaling molecules in muscle cells, such as p38 MAPK, ERK1/2, CREB and AMPK (Kim et al., 2018). On the other hand, the canonical signaling pathway cAMP/PKA seems to be important to maintain muscle mass under basal conditions by restraining FoxO transcriptional activity and the expression of multiple components of the UPS and ALS (Silveira et al., 2020). Moreover, these β_2_-AR agonists also induce a shift in muscle composition from slow to fast fiber type (Kim et al., 2018; Gonçalves et al., 2019). Thus, although RE training and pharmacological stimulation of β_2_-ARs alone promote muscle remodeling and adaptation, the physiological role of endogenous catecholamines and β_2_-ARs in mediating muscle anabolism to RE remains unknown. Therefore, the current study was undertaken to investigate the effects of the β_2_-ARs blockade on the acute molecular changes induced by a single bout of RE in rodent skeletal muscles. By using a pharmacological approach, we demonstrate that the β_2_-AR activation during RE is required to induce *Nr4a3* and to prevent the overexpression of atrophic genes such as *Mstn*, *Murf1/Trim63*, and *Atrogin-1/Mafbx32.*

## 2 Material and methods

### 2.1 Animals and treatments

C57Bl6/J mice (8-week-old male mice, ∼20–23 g) were housed in a room with a 12–12 h light–dark cycle (starting at 6:00 a.m.) and given free access to water and a normal lab chow diet (Nuvilab-CR1; Nuvital, Curitiba, PR, Brazil) until the start of the experiments. The sample size used for each experiment is indicated in the figure legends. The animals were randomly divided into two or three groups as follows: control (CON) and resistance exercise (RE) (experiment one); or CON, RE and RE under β_2_-AR blockade (ICI + RE) (experiment two). Mice from ICI + RE group were treated intraperitoneally (i.p.) with 10 mg kg^−1^ of the selective β_2_-AR antagonist (ICI 118,551; Sigma-Aldrich) 30 min before the RE bout (Azevedo Voltarelli et al., 2021), whereas mice from CON and RE groups were injected with vehicle (0.9% saline). Immediately after (0 h) and 3 hours (3 h) after exercise cessation, the animals were anesthetized with isoflurane by using the open-drop technique (Risling et al., 2012) and euthanized by decapitation with the aid of a rodent guillotine for collecting blood and muscle samples (experiment one) or by cervical dislocation when collecting muscle samples only (experiment two). For this, when the animal was fully anesthetized, the thumb and index finger were positioned on either side of the neck at the base of the skull. With the other hand, the base of the tail was quickly pulled, causing separation of the cervical vertebrae from the skull. After euthanasia, muscles were rapidly harvested, weighed, frozen in liquid nitrogen, and stored at −80°C until further analyses. All experiments were performed accordingly to the ethical principles for animal research adopted by the Brazilian College of Animal Experimentation and were approved by the Ribeirão Preto Medical School from the University of São Paulo - Commission of Ethics in Animal Research (no. 207/2014).

### 2.2 Experimental design

As above mentioned, the present study was divided into two experiments. Experiment one was performed to test whether the acute RE protocol would be able to increase sympathetic activity. For that, the animals were randomly divided into two groups: 1) CON and 2) RE groups. After the familiarization period with the ladder apparatus for RE and the maximum voluntary carrying capacity (MVCC) test, mice from the RE group were subjected to a single bout of RE (see *Resistance Exercise Protocol* section). Immediately after (0 h) the exercise session, the animals were anesthetized with isoflurane and euthanized by decapitation for collecting blood and muscle samples in order to analyze plasma catecholamines by HPLC and muscle proteins content, and phosphorylation by immunoblotting. Experiment two was performed to determine the contribution of β_2_-ARs to skeletal muscular transcriptional response following a single bout of RE. For that, the animals were randomly divided into three groups: 1) CON, 2) RE, and 3) ICI + RE. After the familiarization period with the ladder apparatus for RE and MVCC test, mice from RE and ICI + RE groups were submitted to the RE protocol. Three hours (3 h) after the exercise session, the animals were anesthetized with isoflurane and euthanized by cervical dislocation, and muscles were rapidly harvested and stored at −80°C until qPCR and Western blotting analysis. The 3 h period was chosen because rodents subjected to a single bout of exercise showed transient changes in mRNA levels in the first hours of recovery, typically between 3h and 12 h after exercise cessation (Egan and Zierath, 2013; Egan and Sharples, 2023). Mice from the CON group remained confined in their cages during both experimental protocols, except when all animals were submitted to the familiarization period with the ladder apparatus for RE and MVCC test.

### 2.3 Resistance exercise protocol

**FIGURE 1:**
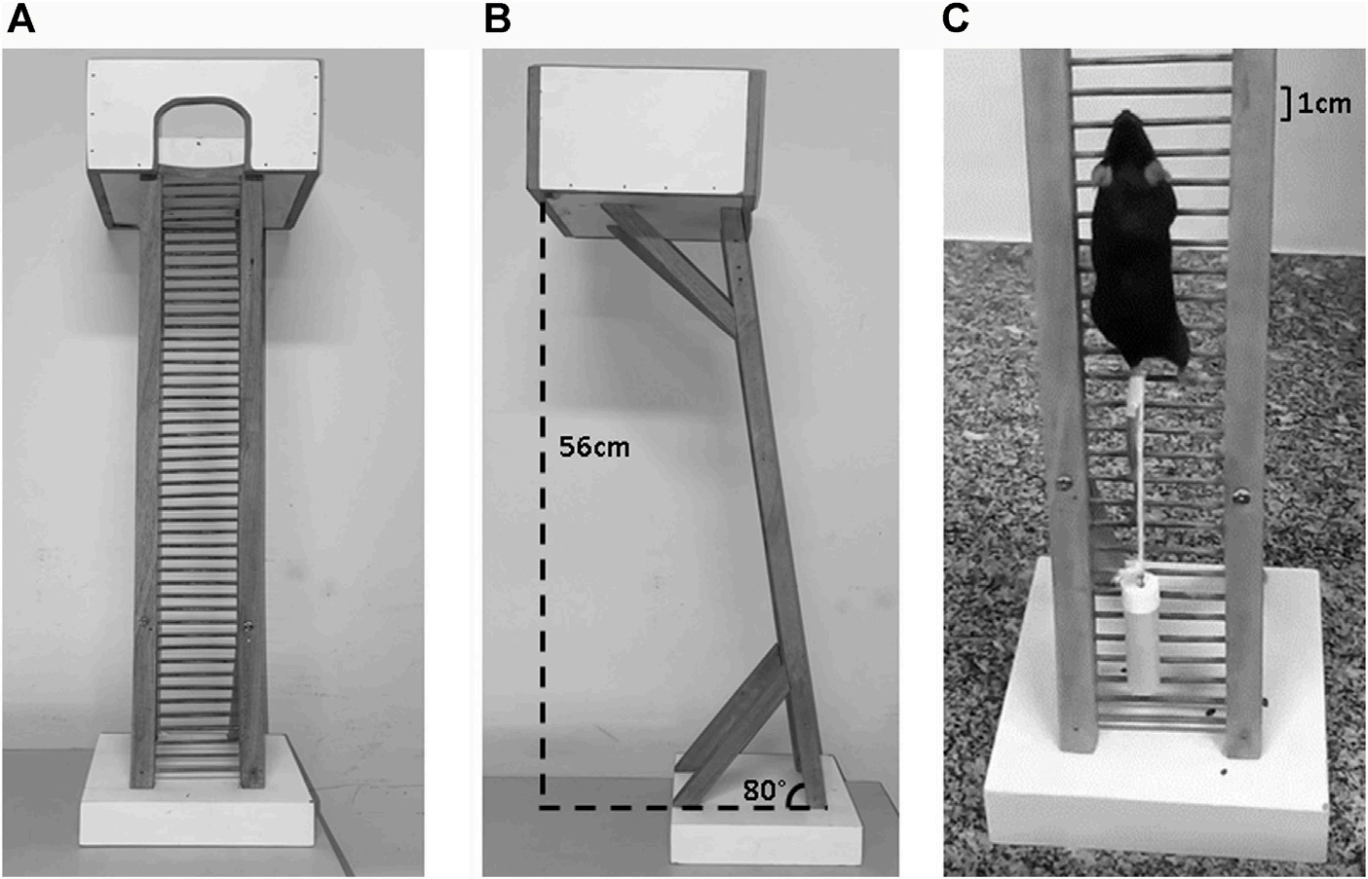
**(A)** The apparatus for resistance exercise (RE) in mice. **(B)** At the top of the ladder is a housing chamber where the mice are allowed to rest between climbs (60 s). **(C)** A mouse is shown climbing a 56 cm, 80° incline ladder with the weight attached to the tail.

The resistance exercise (RE) protocol was based on previous reports (Frajacomo et al., 2015; Padilha et al., 2019) and consisted of ladder climbing (80° incline; 1.0 cm space between steps, and 56 cm height) using progressive overload (see Figures 1A–C). Initially, all mice were familiarized to voluntary climbing the ladder over five consecutive days. For this, mice were positioned on the base of the ladder and encouraged to climb by pushing them to initiate movements. These stimuli were applied until each animal was able to complete an entire climb (Padilha et al., 2017). Forty-8 h after the last familiarization session, the animals were submitted to MVCC test, which consisted of carrying loads equivalent to 50%, 75%, 90%, and 100% of body mass attached to the tail of each mouse. When the animal reached the covered chamber (Figure 1A), an interval of 60 s was given between each climbing bout. After climbing the ladder with 100% of its body mass, a 3 g load was added for the next climbs until a load that incapacitated the animal to fully climb the ladder length. The maximum load (MAX) achieved in the MVCC test was used as a parameter to determine the carrying load during the acute RE session. Finally, 48 h after MVCC test, only the animals from RE and ICI + RE groups were submitted to MAX-based protocol of acute RE (50% of MAX, 75%, 90%, 100%, 100% plus 3 g until failure). Failure was defined as the incapacity to carry the weight even after two successive attempts. All animals remained food deprived from the start of the experiments until euthanasia (about 3.5 h).

### 2.4 Catecholamines and corticosterone levels

Both plasma catecholamines, epinephrine (EPI) and norepinephrine (NE), and the tissue NE from *gastrocnemius* (GAS) and *tibialis anterior* (TA) muscles were assayed as previously described (Garofalo et al., 1996) using HPLC (LC-7A, Shimadzu Instruments) with a 5-μm Spherisorb ODS-2 reversed-phase column (Sigma-Aldrich). Serum corticosterone levels were measured by specific radioimmunoassay as previously described (Durlo et al., 2004).

### 2.5 Western blotting analysis

GAS and TA muscles were homogenized in RIPA buffer containing 10 mM sodium pyrophosphate, 100 mM sodium fluoride, 10 mM sodium orthovanadate, 5 μg mL^−1^ of aprotinin, 1 mg mL^−1^ of leupeptin, and 1 mM phenylmethyl-sulfonyl fluoride (PMSF). Lysates were subjected to sodium dodecyl sulfate-polyacrylamide gel electrophoresis (SDS-PAGE) and immunoblotted using antibodies listed in Table 1. Primary antibodies were detected using peroxidase-conjugated secondary antibodies and visualized using enhanced chemiluminescence (ECL) reagents on ChemiDoc XRS + System (Bio-Rad). Band intensities were quantified using the software ImageJ/Fiji (version 1.52 d, National Institutes of Health, United States).

**TABLE 1:** Antibodies for Western blot.

Protein	Dilution for WB	Manufacturer
phospho-Ser^133^ CREB	1:2000	Cell Signaling
phospho-Ser/Thr PKA	1:1000	Cell Signaling
phospho-Thr^172^ AMPK	1:1000	Cell Signaling
phospho-Ser^256^ FoxO1	1:1000	Cell Signaling
phospho-Thr^24^ FoxO1	1:1000	Cell Signaling
phospho-Thr^32^ FoxO3	1:1000	Cell Signaling
phospho-Thr^202^ ERK1	1:2000	Cell Signaling
phospho-Tyr^204^ ERK2	1:2000	Cell Signaling
phospho-Thr^180^/Tyr^182^ p38-MAPK	1:1000	Cell Signaling
phospho-Ser^473^ AKT	1:1000	Cell Signaling
phospho-Ser^235/236^ S6	1:1000	Cell Signaling
phospho-Ser^240/244^ S6	1:1000	Cell Signaling
phospho-Ser^21^ GSK3α	1:1000	Cell Signaling
phospho-Ser^9^ GSK3β	1:1000	Cell Signaling
phospho-Thr^286^ CaMKII	1:1000	Cell Signaling
Atrogin-1	1:1000	Santa Cruz
α- Tubulin	1:2000	Santa Cruz
β-actin	1:2000	Santa Cruz

### 2.6 Real-time qPCR

The analysis of qPCR was performed at the Laboratory of Metabolism Control from Ribeirão Preto Medical School (University of São Paulo). After the exercise protocol described above, GAS muscles were immediately frozen in liquid nitrogen and stored at −80°C. Total RNA was extracted from muscle using TRIzol (50 mg of muscle was added to 0.5 mL of TRIzol, Invitrogen^®^). Samples were homogenized in tubes using a TissueLyser II (Qiagen^®^) with 5 mm stainless steel beads for 2 × 1 min cycles at 30 Hz, resting on ice in between. Homogenates were cleared by centrifugation at 10,000 x g for 5 min at 4°C. RNA extraction was performed according to TRIzol manufacturer’s instructions (Invitrogen^®^). RNA was eluted in 50 μL of RNase-free water and stored at − 80°C. RNA samples were treated with DNase I, RNase-free (Thermo Fisher Scientific^®^), to remove genomic DNA contamination. RNA samples were quantitated using NanoDrop One spectrophotometer (Thermo Fisher Scientific^®^), following the manufacturer’s instructions. The same device was used to assess the purity of RNA by measuring 260/280 and 260/230 ratios of absorbance values. Samples presenting a 260/280 ratio of ∼2 and 260/230 ratio of 2–2.2 were accepted as “pure” for RNA. According to the manufactures’ protocols, 1 μg of RNA was reverse transcribed into cDNA using 0.5 μL of SuperScript IV First-Strand Synthesis System (Invitrogen^®^). cDNA was diluted 25-fold with nuclease-free water. For qPCR, the total volume per reaction was 10 μL containing 5 μL of cDNA (2 ng/μL), 4.8 μL of PowerUp SYBR Green Master Mix (Thermo Fisher^®^), and 0.2 μL of primers (forward and reverse mixture; 50 μmol/L stock; Table 2). qPCR run on Applied Biosystems™ 7,500 Real-Time PCR System, using the recommended cycling conditions as follows: a pre-incubation of 2 min at 50°C and 10 min at 95°C, followed by a two-step amplification program of 40 cycles set at 95 °C for 15 s (denaturation) and 60 °C for 1 min (annealing + extension) and, finally, a dissociation stage set at 95 °C for 15 s, 60 °C for 1 min and 95 °C for 15 s. The last stage was performed to evaluate the quality of qPCR reactions regarding of nonspecific amplification and primer-dimer formation in a dissociation curve for each gene. The amplification specificity for each primer was confirmed by observing the single melt curve peak after the completion of qPCR. Primer sequences were designed utilizing Primer3Plus (https://www.primer3plus.com/) in conjunction with OligoAnalyzer 3.1 (https://eu.idtdna.com/site) and cross-referenced using the Basic Local Alignment Search Tool program (https://blast.ncbi.nlm.nih.gov/Blast.cgi). A six-point relative standard curve was prepared for each gene by using five-fold serial dilutions of pooled cDNA samples in duplicate. No threshold cycle quantification value for the no template control was detected. The relative expression levels of target genes were calculated using the 2^−ΔΔCT^ method (Schmittgen and Livak, 2008). Data from the target genes were normalized by the expression of *Rpl39* as a reference gene.

**TABLE 2:** Oligonucleotide primers used for qPCR analysis.

Gene	Forward primer (5′-3′)	Reverse primer (3′-5′)
*Ppargc1α*	AAT​CCA​GCG​GTC​TTA​GCA​CT	TTT​CTG​TGG​GTT​TGG​TGT​GA
*Sik1*	TCC​ACC​ACC​AAA​TCT​CAC​CG	GTT​TCG​GCG​CTG​CCT​CTT​C
*Map1lc3b*	CGT​CCT​GGA​CAA​GAC​CAA​GT	TCC​GTC​CTT​CGC​TTC​ATA​GG
*Gabarapl1*	CAT​CGT​GGA​GAA​GGC​TCC​TA	ATA​CAG​CTG​GCC​CAT​GGT​AG
*Ctsl*	CAT​CGT​GGA​GAA​GGC​TCC​TA	ATA​CAG​CTG​GCC​CAT​GGT​AG
*Bnip3*	TTC​CAC​TAG​CAC​CTT​CTG​ATG​A	GAA​CAC​CGC​ATT​TAC​AGA​ACA​A
*Nr4a1*	AGC​TTG​GGT​GTT​GAT​GTT​CC	AAT​GCG​ATT​CTG​CAG​CTC​TT
*Nr4a3*	TGC​AGA​GCC​TGA​ACC​TTG​AT	TTA​ACC​CAT​GTC​GCT​CTG​TG
*Mstn*	TTG​CAA​AAT​TGG​CTC​AAA​CAG​C	AAG​GGA​TTC​AGC​CCA​TCT​TCT​C
*Igf-1*	CTC​AGA​CAG​GCA​TTG​TGG​ATG​AGT	GGT​CTT​GTT​TCC​TGC​ACT​TCC​TCT
*Il-6*	TAG​TCC​TTC​CTA​CCC​CAA​TTT​CC	TTG​GTC​CTT​AGC​CAC​TCC​TTC
*Murf1*	TGTGCAAGGAACACGAAG	TGAGAGATGATCGTCTGC
*Atrogin-1*	GCAGAGAGTCGGCAAGTC	CAGGTCGGTGATCGTGAG
*Adr2*	GAG​CAC​AAA​GCC​CTC​AAG​AC	TGG​AAG​GCA​ATC​CTG​AAA​TC
*Myh1*	ACC​TTG​TGG​ACA​AAC​TGC​AA	AGC​TTG​TTG​ACC​TGG​GAC​TC
*Myh2*	TTG​GTG​GAT​AAA​CTC​CAG​GC	CAG​CTT​GTT​GAC​CTG​GGA​CT
*Myh4*	ACA​GAC​TAA​AGT​GAA​AGC​CTA​CAA	CAC​ATT​TTG​TGA​TTT​CTC​CTG​TCA​C
*Myh7*	AGC​AGG​AGC​TGA​TTG​AGA​CC	TGT​GAT​AGC​CTT​CTT​GGC​CT
*Rpl39*	TCC​TGG​CAA​AGA​AAC​AAA​AGC	TAG​ACC​CAG​CTT​CGT​TCT​CCT

### 2.7 Statistics

The distribution and variance homogeneity were tested using Shapiro-Wilk test. The data are expressed as means ± standard error mean (SEM). According to each experimental design, non-paired Student’s t-test or one-way analysis of variance (ANOVA) followed by Tukey’s *post hoc* were used in normally distributed variables or those that showed a normal distribution after log transformation. Kruskal–Wallis test followed by Dunn’s *post hoc* test was used for non-parametric data. Data were analyzed using JASP 0.16.2 software (GNU Affero GPL v3 license, Department of Psychological Methods, University of Amsterdam) and GraphPad Prism version 7.0 (Graph Pad Softwares, San Diego, CA, United States). The significance level adopted was 5% (*p* ≤ 0.05).

## 3 Results

### 3.1 A single bout of RE acutely increases the circulating levels of norepinephrine and corticosterone

**FIGURE 2:**
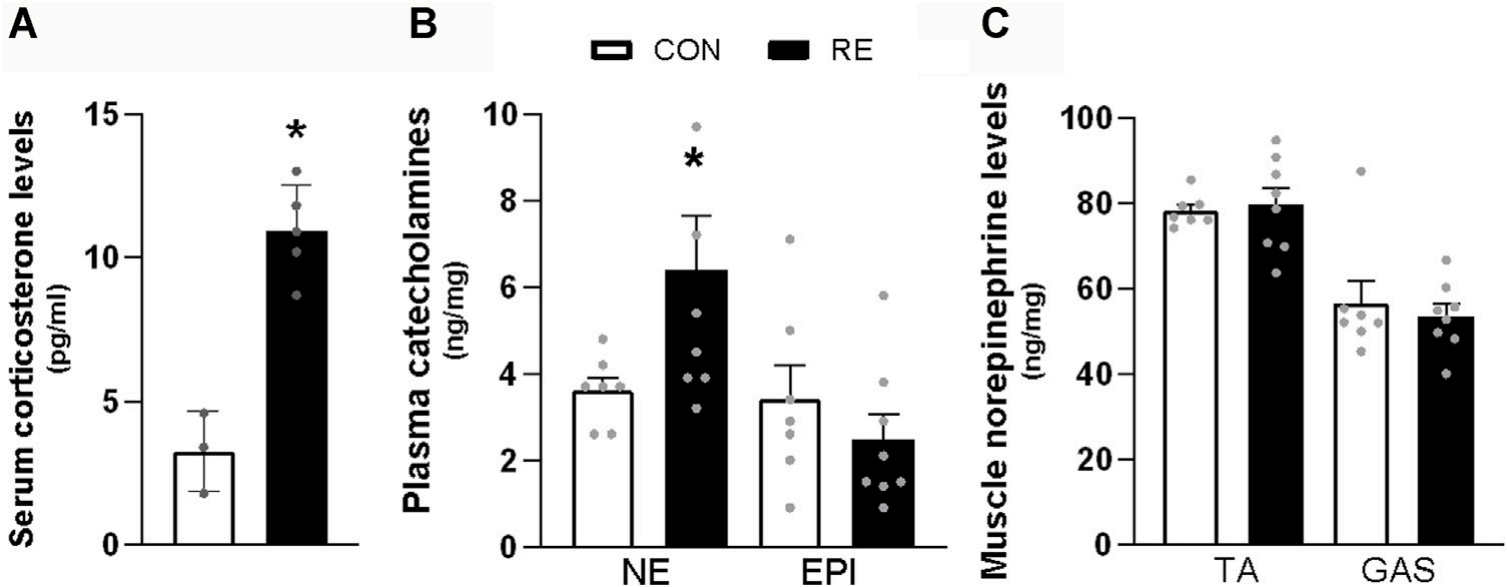
Acute effect of a single bout of resistance exercise (RE) on the activation of sympathetic-adrenomedullary and the hypothalamus-pituitary-adrenal (HPA) axis of mice. **(A)** Serum corticosterone, **(B)** plasma catecholamines, and **(C)** content of norepinephrine in *tibialis anterior* (TA) and *gastrocnemius* (GAS) muscles of mice, immediately after (0 h) the RE session. Data are presented as mean ± SEM of 7-8 mice. (*p ≤ 0.05 vs CON group, Student’s t-test). NE, norepinephrine; EPI, epinephrine.

Since it has been demonstrated that RE may stimulate a stress response by activating both the sympathetic-adrenomedullary and the hypothalamus-pituitary-adrenal (HPA) axis (Kraemer and Ratamess, 2005; Athanasiou et al., 2023), we first investigated the acute effect of the ladder climbing-based RE protocol on circulating catecholamines and corticosterone levels, immediately after (0 h) RE cessation. As expected, a single bout of RE acutely increased the serum corticosterone (∼3-fold) and plasma NE (∼2-fold) levels (Figures 2A, B). On the other hand, plasma EPI levels were not altered (Figure 2A). Moreover, because muscle sympathetic nerve activity (MSNA) has also been reported to increase during exercise (Katayama and Saito, 2019), we evaluated the NE levels in GAS and TA muscles of mice immediately after (0 h) the RE cessation. As shown in Figure 2C, the NE levels in both muscles were unchanged following RE.

### 3.2 A single bout of RE acutely stimulates intracellular pathways involved in energy metabolism and adrenergic signaling in skeletal muscle

**FIGURE 3:**
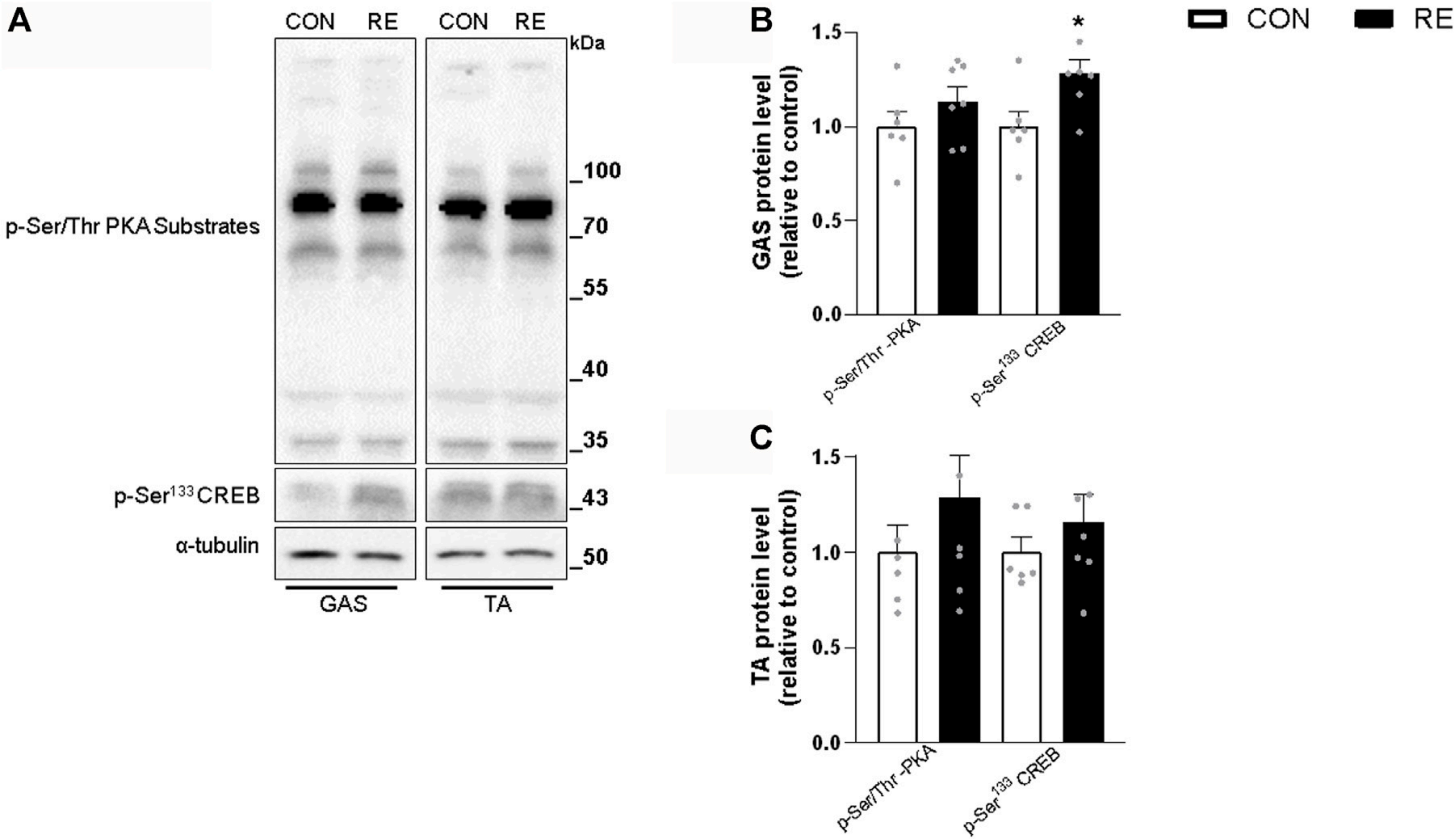
Acute effect of a single bout of resistance exercise (RE) on the PKA/CREB signaling pathway in skeletal muscle from mice. **(A)** Representative western blots of phosphorylation levels of PKA substrates and CREB in muscles of exercised mice, immediately (0 h) after the RE session. **(B, C)** Densitometric and statistical analysis of the p-Ser/Thr PKA substrates and p-Ser^133^ CREB protein content in **(B)** *gastrocnemius* (GAS) and **(C)** *tibialis anterior* (TA) muscles. Phosphorylated proteins were normalized to α-tubulin. Data are presented as mean ± SE of 6-7 muscles. (*p ≤ 0.05 vs CON group, Student’s t-test).

**FIGURE 4:**
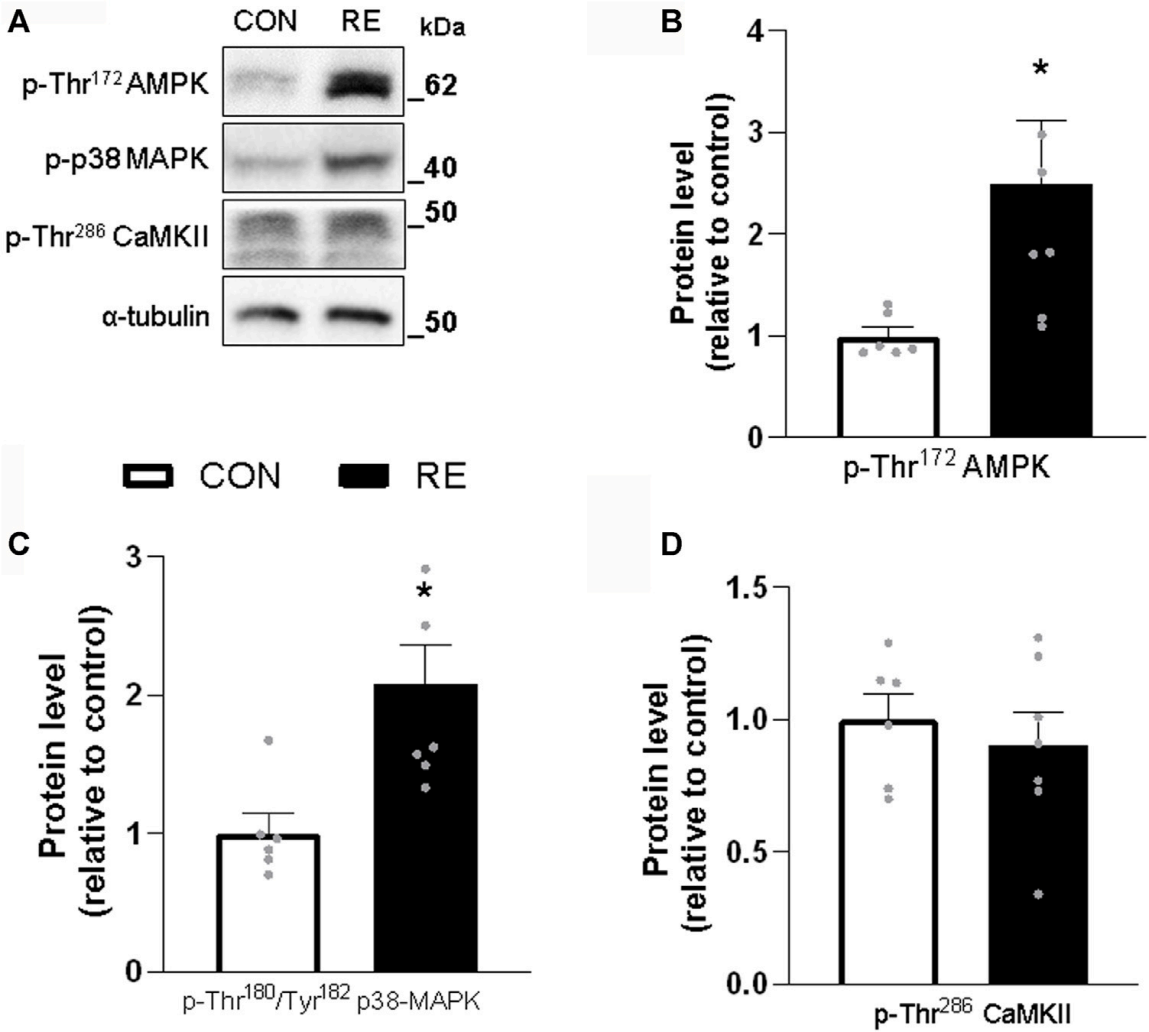
Acute effect of a single bout of resistance exercise (RE) on the activation of AMPK, p38 MAPK and CaMKII signaling in muscles of exercised mice. **(A)** Representative western blots of phosphorylation levels of AMPK, p38 MAPK and CaMKII in *gastrocnemius* (GAS) muscles of exercised mice, immediately after (0 h) the RE session. **(B–D)** Densitometric and statistical analysis of the **(B)** p-Thr^172^AMPK, **(C)** p-Thr^180/Tyr182^ p38 MAPK and **(D)** p-Thr^286^ CaMKII protein content presented in Figure 4A. Phosphorylated proteins were normalized to α-tubulin. Data are presented as mean ± SEM of 6-7 muscles. (*p ≤ .05 vs CON group, Student’s t-test).

**FIGURE 5:**
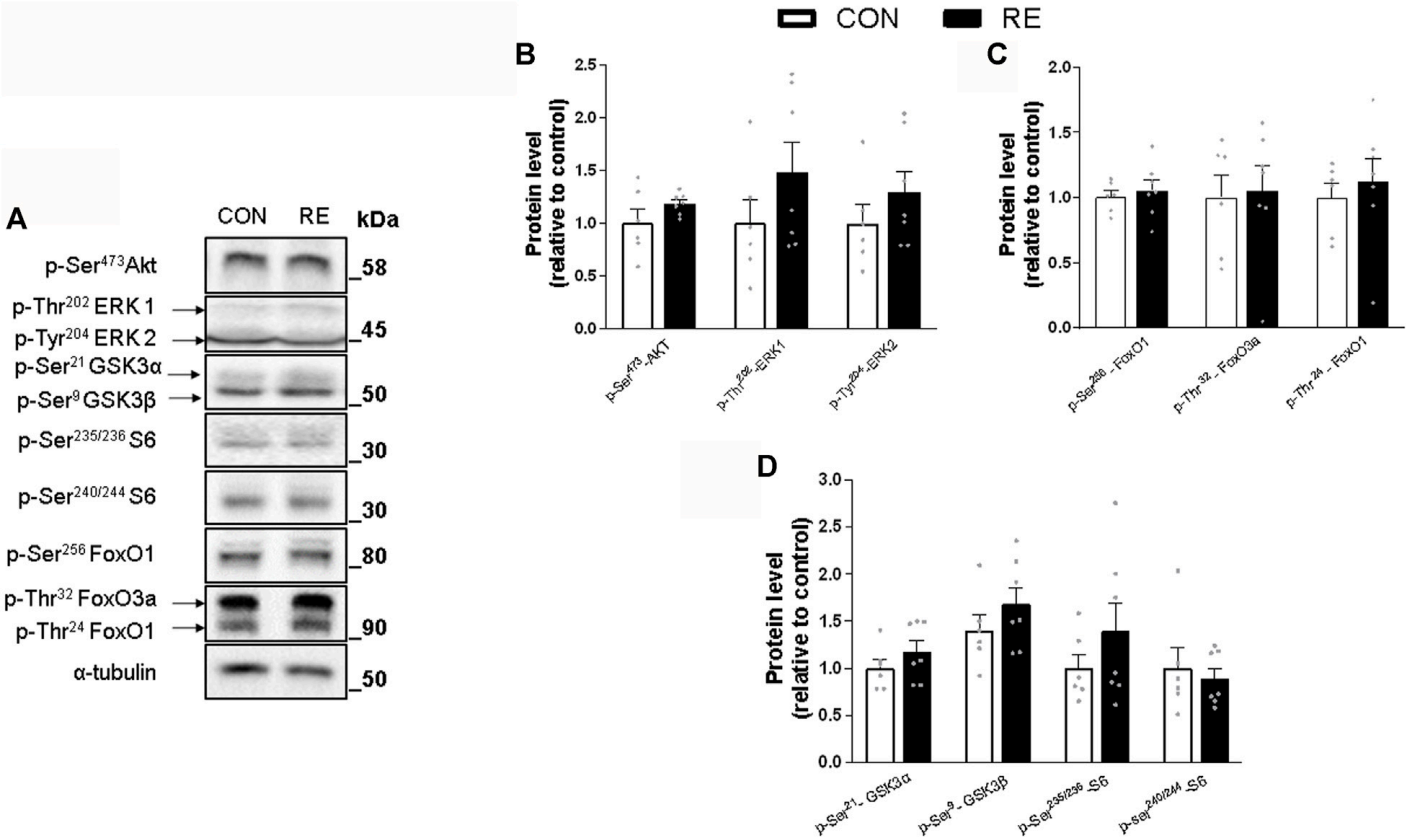
Acute effect of a single bout of resistance exercise (RE) on the Insulin/IGF-1 signaling in muscles of exercised mice. **(A)** Representative Western blot of phosphorylation levels of Akt, ERK1/2, GSK3, S6, FoxO1 and FoxO3 in *gastrocnemius* (GAS) muscles of exercised mice, immediately after (0 h) the RE session. **(B–D)** Densitometric and statistical analysis of the **(B)** Akt and ERK/12; **(C)** FoxO1 and FoxO3a; and **(D)** GSK3 and S6 protein content. Phosphorylated proteins were normalized to α-tubulin. Data are presented as mean ± SEM of 6-7 muscles. (*p ≤ 0.05 vs CON group, Student’s t-test).

Because RE stimulated systemic SNS activity, we examined whether the canonical *ß*-AR signaling pathway (*i.e.,* PKA/CREB) was also activated in the muscles of exercised mice immediately after (0 h) the RE cessation. Although TA muscles were unaffected, a single bout of RE acutely increased the phosphorylation levels of CREB (∼30%) in GAS muscles, without altering the phosphorylation levels of other PKA substrates at Ser/Thr (Figures 3A–C). These findings suggest that the activity of transcription factor CREB was increased, but other upstream kinases than PKA may have also been responsible for such an effect. In addition to PKA, the protein kinases AMPK, CAMKII, and p38 MAPK (via mitogen-and stress-activated kinase 1; MSK1), may induce CREB phosphorylation at Ser^133^ (Shaywitz and Greenberg, 1999; Bengal et al., 2020). Indeed, we observed a substantial increase in the phosphorylation levels of p38 MAPK (2-fold) and AMPK (2-fold), but not in CaMKII, in GAS muscles (Figures 4A–D). On the other hand, we did not observe any significant difference in phosphorylation status of main signaling pathways controlling muscle protein synthesis (i.e., Akt, ERK1/2, and their downstream targets GSK-3 and S6), and protein degradation (i.e., FoxO1 and FoxO3a), when assessed immediately after exercise cessation (Figures 5A–D). Together, these data indicate that a single bout of RE acutely stimulates plasma NE and CREB phosphorylation in muscles, probably via activation of the p38 MAPK and/or AMPK pathways (Thomson et al., 2008).

### 3.3 *β*_*2*_ *-*adrenoceptor blockade reduces the CREB-target genes expression in muscles from exercised mice

**FIGURE 6:**
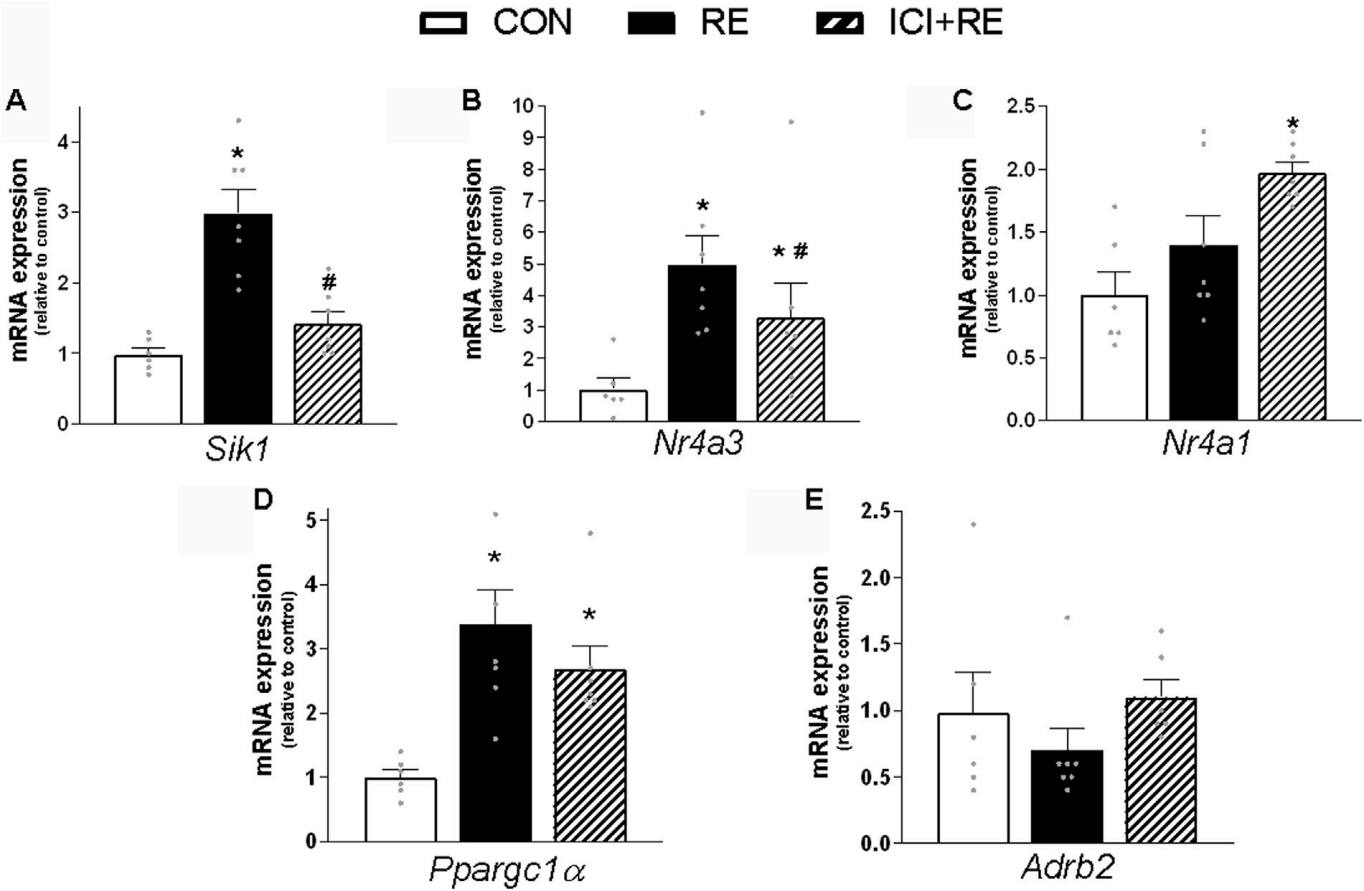
Effect of the β_2_-AR blockade on CREB-target genes expression in skeletal muscle of exercised mice. Three hours (3 h) after the exercise session, the **(A)** *Sik1*; **(B)** *Nr4a3;* **(C)** *Nr4a1*; **(D)** *Ppargc1α* and **(E)** *Adrb2* mRNA levels were analyzed in *gastrocnemius* (GAS) muscles from exercised mice pre-treated with β_2_-AR antagonist ICI 118,551 (single dose - 10 mg kg^−1^, i. p., 30 min prior RE). Data are presented as mean ± SEM of 6-7 muscles. (*p ≤ 0.05 vs CON group, #*p* < 0.05 vs RE group, bidirectional AVONA test).

**FIGURE 7:**
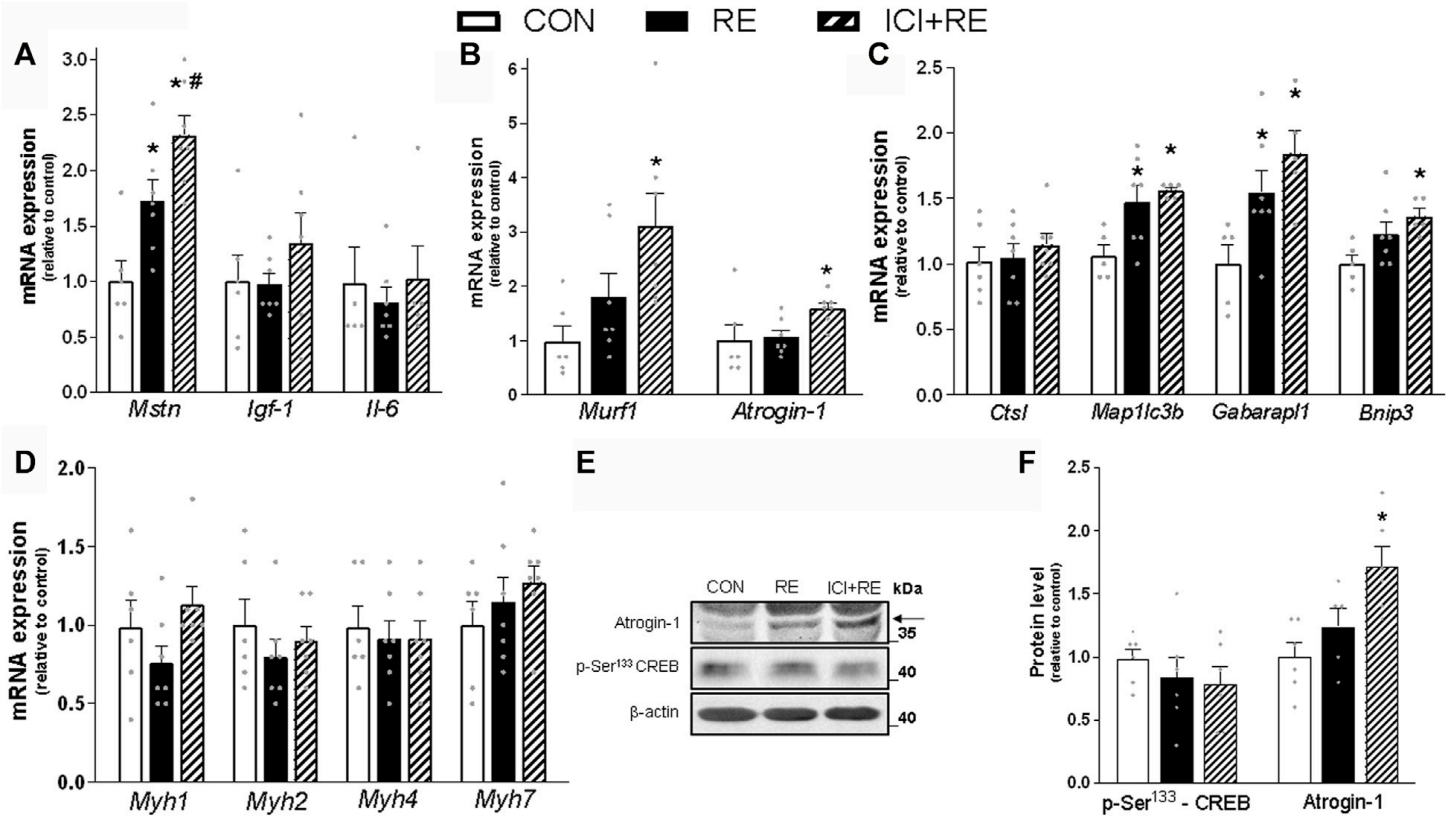
Effect of the β_2_-AR blockade on the mRNA levels of genes involved in metabolism and plasticity in muscles of exercised mice. Three hours (3 h) after the exercise session, the **(A)** *Mstn*, *igf-1* and *Il-6* (myokines); **(B)** *Murf1* and *Atrogin-1* (atrophy-related genes); and **(C)** *Ctsl*, *Map1lc3b*, *Gabarapl1* and *Bnip3* (autophagy-related genes); **(D)** *Myh1*, *Myh2*, *Myh4* and *Myh7* mRNA levels were analyzed in *gastrocnemius* (GAS) muscles of exercised mice pre-treated with β_2_-AR antagonist ICI 118,551 (single dose; 10 mg kg^−1^, i. p., 30 min prior RE). **(E)** Representative western blots of phosphorylation levels of CREB and protein content of Atrogin-1 in gastrocnemius (GAS) muscles of exercised mice, three hours (3 h) after the exercise session. **(F)** Densitometric and statistical analysis of the protein content presented in **(E)**. Phosphorylated proteins were normalized to β-actin. Data are presented as mean ± SEM of 6-7 muscles. (*p ≤ 0.05 vs CON group, #*p* < 0.05 vs RE group, bidirectional AVONA test).

Because most of the metabolic actions of catecholamines in skeletal muscle are exerted through PKA/CREB signaling pathway (Berdeaux and Stewart, 2012), we hypothesize that the acute increase in plasma NE levels induced by RE would lead to enhanced muscle transcriptional activity of CREB through a β_2_-AR-dependent mechanism. To test this hypothesis, we first subjected mice to the maximum voluntary carrying capacity (MVCC) test under β_2_-AR blockade with the selective β_2_-AR antagonist ICI 118,551 (ICI; 10 mg kg^−1^, i. p., 30 min prior RE session), in order to evaluate whether ICI pre-treatment would affect mice performance. As shown in Table 3, the mean values of the ICI + RE group were very similar to saline treated-group (41.7 ± 1.1 and 42.8 ± 0.7g, for maximal carrying load; 9.0 ± 1.2 and 10.0 ± 0.7 for number of climbs until exhaustion; 290.69 ± 23.7, and 326.8 ± 24,3 g for training volume; and 16.0 ± 0.9 14.5 ± 1.2min for duration of RE session). Thereafter, we measured the phosphorylation levels of CREB and the mRNA expression of its specific target genes (*i.e*., *Sik1, Nr4a3, Nr4a1, Ppargc1a* and *Adrb2*), 3 h after the exercise cessation. As shown in Figure 6, *Sik1* (∼3-fold), *Nr4a3* (∼6-fold) and *Ppargc1a* (∼3.5-fold) mRNA levels were upregulated in GAS muscles 3 h after acute RE, even though CREB phosphorylation returned to the basal values (Figures 7E, F). Performing RE under β_2_-AR blockage (ICI + RE) resulted in reduced levels of *Sik1* (Figure 6A) and *Nr4a3* mRNA expression (∼50%; Figure 6B), when compared with RE alone. On the other hand, ICI + RE did not alter the mRNA expression of *Ppargc1a* (Figure 6D). Interestingly, the *Adrb2* mRNA was not affected by acute RE or ICI + RE (Figure 6E). Thus, our data suggest that acute RE requires the activation of β_2_-AR to regulate the muscle expression of specific CREB-target genes, raising the possibility that at least part of this effect might be directly mediated by plasma NOR.

**TABLE 3:** Body mass and maximum voluntary carrying capacity (MVCC) data.

Parameter	CON	RE	RE + ICI
Body mass (g)	21.6 ± 0.7	21.8 ± 1.49	21.1 ± 0.6
Maximal load (g)	43.7 ± 1.3	41.7 ± 1.1	42.8 ± 0.7
Number of climbs	-	9.0 ± 1.2	10.0 ± 0.7
Training volume (g)	-	290.69 ± 23.7	326.8 ± 24.3
Protocol time (min)	-	16.0 ± 0.9	14.5 ± 1.2

All values are presented as means ± standard error of the mean; CON: control group; RE: resistance exercise group; ICI + RE: resistance exercise under β_2_-AR, blockade group.

### 3.4 *β*_*2*_ *-*AR blockade stimulates the expression of atrophic genes in muscles from exercised mice

Because we have shown that catecholamines and β_2_-AR agonists induce anti-catabolic effects on skeletal muscle protein metabolism (Graça et al., 2013; Gonçalves et al., 2019) we next investigated the role of β_2_-AR activation in the expression of myokines and UPS- and ALS-related genes 3 h after a single bout of RE. As shown in Figure 7A, the mRNA levels of the negative regulator of muscle mass myostatin (*Mstn*; ∼70%), but not the growth factor *igf-1* or the cytokine *Il-6*, was increased by a single bout of RE in GAS muscles. Moreover, acute RE only slightly increased the mRNA expression of *Map1lc3b* (∼30%) and *Gabarapl1* (∼50%), an effect that was not observed in *Ctsl* and *Bnip3* (Figures 7B, C). In contrast, the β_2_-AR blockade increased the gene expression of *MuRF1/Trim63*, (∼3.5-fold), *Atrogin-1/Mafbx32* (∼50%), and *Bnip3* (∼40%) (Figures 7B, C), as well as the protein content of Atrogin-1 (∼80%) (Figure 7E). Importantly, the increase in *Mstn* mRNA expression was higher (∼40%) in GAS muscles from ICI + RE, when comparted to RE group (Figure 7A). Because both chronic treatment with β_2_-AR agonists and RE training induce a shift in muscle composition from slow to fast phenotype (Mounier et al., 2007), we also measured the mRNA levels of the genes that encodes for myosin heavy chain (MHC) isoforms MHC-I, MHCIIA MHCIIX and MHCIIB (*Myh7, Myh2, Myh1 and Myh4*, respectively). As shown in Figure 7D, neither RE nor ICI + RE affected the mRNA levels of these genes. Altogether, these data indicate that the β_2_-AR activation decreases the expression of atrophic genes after a single bout of RE.

## 4 Discussion

The present study provides molecular evidence that β_2_-AR activation during a single bout of RE upregulates the *Nr4a3* gene*,* a central regulator of the acute RE response, and downregulates the expression of atrophic genes (i.e., *Mstn*, *Murf1/Trim63*, *Atrogin-1/MaFbxo32,* and *Bnip3*) in mouse skeletal muscle after the exercise session. These data provide further evidence that the SNS may be physiologically important to muscle adaptations in response to resistance training. Here, we demonstrated that mice performing a single bout of a ladder climbing-based RE protocol showed a marked increase in plasma NE levels, without significant change in plasma EPI. The increase in plasma catecholamines was expected, because exercise is considered a stressful stimulus that stimulates the sympathoadrenal system to meet the physiological demand of active skeletal muscles (Athanasiou et al., 2023). Although both plasma NE and EPI levels continuously increase as a function of RE intensity (Kraemer and Ratamess, 2005), the plasma NE response is greater when compared to EPI (Greiwe et al., 1999). Similar to plasma catecholamines, muscle sympathetic nerve activity (MSNA) gradually increases in proportion to the rise in exercise intensity (Katayama and Saito, 2019). More important, we and others have recently demonstrated that the SNS directly innervates neuromuscular junctions and regulates skeletal muscle metabolism, fiber type composition, and cross-sectional area in normal conditions (Khan et al., 2016; Rodrigues et al., 2019). In the present study, however, the increase in muscle content of NE was not observed after the RE session, most likely due to NE diffusion from synapses (mainly from active muscles) into the plasma (Rowell and O’Leary, 1990). Indeed, plasma NE concentration is greatly influenced by the rate of NE diffusion. Therefore, it cannot be excluded that an increase of NE release directly into muscle may have occurred during the RE. The measurement of muscle NE turnover (Graça et al., 2013) and muscle sympathetic denervation (Silveira et al., 2014) would be necessary to determine the direct contribution of muscle sympathetic activity to the molecular changes induced by acute RE.

Independently of the systemic or local origin of NE, we have pieces of evidence that SNS stimulates muscle cells in response to RE. The finding in GAS muscle that a single bout of RE increased the phosphorylation levels of CREB, a well-established target of PKA, and its specific target genes (i.e., *Sik1*, *Nr4a3*, and *Ppargc1a*), suggests that the canonic β_2_-AR/PKA/CREB signaling pathway were stimulated. Consistently, the β_2_-AR blockage downregulated the *Sik1* and *Nr4a3* gene expression in GAS muscles from exercised mice. Similar findings were reported by Bruno et al. (Bruno et al., 2014) showing that the expression of *Sik1* mRNA was markedly induced by a high-intensity exercise, and this response was prevented by the pre-treatment with propranolol, a pan *ß*-AR antagonist (Bruno et al., 2014; Goode et al., 2016). However, a few studies have investigated the role of the protein kinase SIK-1 on skeletal muscle physiology. Berdeaux et al. (Berdeaux et al., 2007) proposed that, under adrenergic stimulation with the *ß*-adrenergic agonist isoproterenol, CREB activates the myogenic program by increasing the amount of SIK-1 in C2C12 cells and adult skeletal muscles. When activated (dephosphorylated form), SIK-1 phosphorylates class II histone deacetylases (HDACs) and indirectly promotes the expression of myocyte enhancer factor 2 (MEF2) target genes (Berdeaux et al., 2007). Accordantly, the overexpression of SIK1 in muscle cells induces nuclear export of HDAC5 and increases MEF-2C transcriptional activity *in vitro* (Takemori et al., 2009). Despite these data, there is no evidence that SIK-1 regulates muscle protein metabolism in response to exercise.

In contrast to our data, Goode et al. (2016) suggested that the increase in *Nr4a3* mRNA after endurance exercise is independent on *ß*-AR signaling, since its expression was not attenuated by the treatment with the *ß*-AR antagonist propranolol. These conflicting results may be due to differences in the experimental design, including pharmacological treatment (selective *versus* non-selective β_2_-AR antagonist) and type of exercise bout (endurance exercise *versus* RE). Despite that, recent evidences have shown that the *Nr4a3* gene was robustly induced by a novel RE model in mice (Cui et al., 2020) and by acute RE in human skeletal muscle (Nader et al., 2014). By using gene ontology to highlight pathways activated by inactivity, aerobic *versus* resistance, and acute *versus* chronic exercise, Pillon et al. (2020) identified *Nr4a3* as the most exercise- and inactivity-responsive gene, and determined its role in the regulation of mitochondrial function and glucose uptake in response to electrical pulse stimulation in human myotubes *in vitro*. Additionally, it has been shown that transgenic overexpression of NR4A3 promotes skeletal muscle hypertrophy, oxidative phenotype, and vascularization in mice (Goode et al., 2016). Although it has already been shown that acute exercise (Goode et al., 2016; Cui et al., 2020) and β_2_-AR stimulation (Berdeaux et al., 2007; Pearen et al., 2009) alone may induce the expression of *Sik1* and *Nr4a3* mRNA, to our knowledge, this is the first study to demonstrate that the pre-treatment with the selective β_2_-AR antagonist downregulates the expression of both genes in muscles from exercised mice. Further studies are needed to reveal the specific role of *Nr4a3* and *Sik-1* expression in mediating the effects of β_2_-AR stimulation on muscle protein metabolism in response to chronic RE training.

The reason for differences between TA and GAS muscles in response to acute RE cannot be accounted for in the present study, but exercised muscles are either under the influence of extrinsic (*e.g.*, neural and hormonal) and intrinsic (*e.g.*, mechanical and metabolic) factors that activate/repress several intracellular signaling pathways (Egan and Zierath, 2013). For example, the finding that CREB phosphorylation was induced only in GAS muscles reinforces the hypothesis that acute RE increased the sympathetic activityprobably by a metaborreflex-induced mechanism (Fisher et al., 2015), since GAS muscle, but not TA, is highly recruited during climbing movements (Lourenço et al., 2020). On the other hand, the fact that the expression of *Ppargc1a* mRNA induced by RE was not affected by β_2_-AR blockage raise the possibility that the expression of this transcription co-activator is under control of intrinsic signals, such as increased intracellular calcium concentration [Ca^2+^]_i_, AMP:ATP ratio and mechanical tension, among others (Egan and Zierath, 2013). Accordingly, these signals may trigger the activation of protein kinases involved in several metabolic processes including AMPK, CaMKII, and p38 MAPK. In agreement with this notion, we demonstrated that the phosphorylation levels of AMPK and p38 MAPK, but not CaMKII, increased in GAS muscle immediately after acute RE. It is important to mention that these kinases seem to be activated by an intensity-dependent manner and may stimulate *Ppargc1a* gene transcription by different regulators, such as activating transcription factor2 (ATF2), myocyte enhancer factor 2 (MEF2), CREB, and HDACs (Wojtaszewski et al., 2000; Rose et al., 2006; Akimoto et al., 2008; Egan and Zierath, 2013). Indeed, it has been shown that p38 MAPK can phosphorylate and activate the ATF2 transcription factor, whereas AMPK can directly phosphorylate CREB during exercise, upregulating *Ppargc1a* mRNA (Akimoto et al., 2008; Thomson et al., 2008). Taken together, these data suggest that acute RE may upregulate *Ppargc1a* expression by multiple muscle intrinsic signals in a β_2_-AR-independent manner.

As previously reported (Silvestre et al., 2017), our ladder climbing-based progressive RE increased serum corticosterone (CORT) levels immediately after exercise, and probably reduced insulin (INS) secretion in exercised mice (Raastad et al., 2000; Kraemer and Ratamess, 2005). During catabolic conditions, such as fasting and type 1 diabetes mellitus, the high levels of glucocorticoids associated with low levels of circulating insulin drive the activation of muscle proteolysis by UPS and ALS in order to support liver gluconeogenesis and the energy requirements of the organism (Hu et al., 2009; Graça et al., 2013). In fact, Hu et al. (2009) have demonstrated that both impaired INS signaling and increased endogenous glucocorticoids are required to stimulate muscle proteolysis by UPS. Thus, reduced activity of the INS/Akt signaling leads to a marked increase in atrophic genes and muscle atrophy via the transcriptional activity of FoxO members (*e.g.,* FoxO1 and FoxO3a) (Schiaffino and Mammucari, 2011). Due to the catabolic nature of the acute exercise, it has been shown that the rate of overall protein degradation and the induction of these atrophic genes also increases in human skeletal muscle immediately after the acute RE, likely to prevent the accumulation of damaged proteins and organelles (Phillips et al., 1997; Louis et al., 2007). However, the effect of a single bout of progressive RE-induced acute molecular changes in rodent skeletal muscle remain poorly explored. The present study shows that a single bout of RE performed until exhaustion did not affect the phosphorylation and activation/inactivation status of the major signaling pathways controlling muscle protein synthesis (*i.e.,* Akt, ERK1/2, and their downstream targets GSK-3 and mTOR), and protein degradation (*i.e.,* FoxO), when assessed immediately after exercise session. These data are controversial because published data have not been consistent on whether these signaling are increased or unaffected in response to acute RE (Bolster et al., 2003; Hamilton et al., 2010; Kido et al., 2016; McIntosh et al., 2023). Again, these conflicting results may be due to differences in the experimental approaches, including exercise protocol, species (rodents *versus* humans), and time point of sample collecting after the exercise bout, among others.

It is well known that the SNS also contributes to the establishment of a catabolic state during acute exercise since catecholamines stimulates the catabolism of glycogen and intramuscular triglyceride through β_2_-AR//PKA/CREB signaling in skeletal muscle (Egan and Zierath, 2013; Bruno et al., 2014). Conversely, we and others have consistently shown that both catecholamines and β_2_-AR agonists exert anabolic and anticatabolic actions on protein metabolism, contributing to the maintenance of skeletal muscle mass under basal and catabolic conditions (Navegantes et al., 1999; Baviera et al., 2008; Graça et al., 2013; Khan et al., 2016; Gonçalves et al., 2019; Rodrigues et al., 2019). Thus, we hypothesized that the activation of β_2_-ARs during acute RE could inhibit the expression of atrophic genes in the skeletal muscle of exercised mice. Accordingly, we demonstrate that performing acute RE under β_2_-AR blockade upregulates the expression of the Ub-ligases *Murf1/Trim63* and *Atrogin-1/Mafbx32*. Although the exact molecular mechanisms cannot be accounted for in the present study, treatment with β_2_-agonists has been suggested to inhibit atrophic gene expression by stimulating Akt/FoxO signaling pathway (Gonçalves et al., 2019; Lynch and Ryall, 2008). Our findings are in partial agreement with other studies showing that chemical (Baviera et al., 2008) or surgical (Graça et al., 2013) sympathectomy exacerbates atrophic genes during type I diabetes and fasting, respectively, apparently by a further decrease in Akt stimulation. An alternative possibility to the suppressive action of SNS in atrophic genes during RE is that cAMP/PKA signaling could mediate such an effect. In agreement with this notion, we have recently shown that muscle-specific overexpression of PKI (PKA inhibitor peptide) decreased the phosphorylation levels of CREB and upregulated the transcriptional activity of FoxO and the mRNA expression of *Atrogin-1* and *MuRF1*, resulting in myofiber atrophy (Silveira et al., 2020). More important, the muscle-specific activating of PKA by the overexpression of PKA catalytic subunit suppressed FoxO transcriptional activity by multiple mechanisms *in vivo*, in addition to FoxO phosphorylation, acetylation, and nuclear export (Silveira et al., 2020), raising the possibility that this cAMP/PKA/FoxO signaling may have mediate this anti-catabolic action. Further studies are required to confirm this hypothesis.

An interesting finding of this study was that performing acute RE under β_2_-AR blockade amplified the expression of *Mstn* mRNA in exercised muscles, suggesting that the β_2_-AR signaling restrains *Mstn* overexpression during RE. Since it has been shown that the expression of a constitutively active form of FoxO1 may upregulate *Mstn* mRNA in differenciated C2C12 myotubes (Allen and Unterman, 2007), it is reasonable to speculate that prevention of the activation of PKA/CREB signaling by ICI in the muscles of exercised mice could exacerbate FoxO activity and consequently *Mstn* expression. Additionally, it is also possible that the downregulation of *N4a3* expression by β_2_-AR blockade could further upregulate the *Mstn* mRNA levels in muscles of exercised mice. This hypothesis is based on findings that reduced endogenous *Nr4a3* mRNA levels induced by stable expression of a NOR-1 (*i.e.,* NR4A3) small interfering RNA in C2C12 cells led to a dramatic upregulation of *Mstn* mRNA expression, whereas *NR4a3* overexpression induced by an expression vector (pSG5-NOR-1) repressed *Mstn* promoter activity and gene expression (Pearen et al., 2009; Goode et al., 2016). Although these findings suggest that β_2_-AR/PKA/CREB signaling may contribute to the adaptive anabolic pathways in response to RE, further experiments are needed to confirm all these hypotheses.

In summary, the present data suggest that β_2_-AR stimulation during acute RE upregulates the expression of the hypertrophic gene *Nr4a3* and restrains the atrophic genes *Mstn*, *Murf1/Trim63*, *Atrogin-1/MaFbxo32* and *Bnip3* in skeletal muscle. These effects may be physiologically important for preventing excessive protein breakdown during muscle contractions and for establishing the anabolic state observed during recovery from acute RE, which may contribute, at least in part, to the muscle adaptations in response to regular RE training.

## Data Availability

The original contributions presented in the study are included in the article/Supplementary materials, further inquiries can be directed to the corresponding author.
